# TB-related technical enquiries received at the Research Institute of Tuberculosis, Japan, during COVID-19

**DOI:** 10.5588/pha.24.0014

**Published:** 2024-09-01

**Authors:** M. Urakawa, A. Yasukawa, S. Hirao, M. Ota, Y. Hatamoto, T. Zama, Y. Nagata, T. Yoshiyama

**Affiliations:** Research Institute of Tuberculosis, Tokyo, Japan.

**Keywords:** epidemiology, tuberculosis, technical assistance, TB enquiries

## Abstract

**SETTING:**

Japan: a low-TB-burden country.

**OBJECTIVE:**

To characterise TB-related technical enquiries received in 2020–2022, and share the lessons learnt.

**DESIGN:**

This was a descriptive study.

**RESULTS:**

We received 1,898 communications, of which 1,447 (40.2 per month) were classified as technical enquiries, 34% fewer than the 2,197 enquiries received in 2017–2019. The enquiry rates were highest for Shimane (4.32/100,000 population) and Yamanashi (2.59/100,000 population) prefectures, and lowest in Ehime (0.00/100,000 population) and Yamagata (0.09/100,000 population) prefectures. The main organisations the enquirers belonged to were local governments (*n* = 989, 68.3%) and healthcare facilities (*n* = 242, 16.7%). The enquirers included medical doctors (*n* = 236, 16.3%), nurses (*n* = 814, 56.3%), and the general public (*n* = 141, 9.7%). The most frequent enquiries were about TB diagnosis and treatment, including laboratory diagnosis (*n* = 442, 30.6%), followed by the regulatory framework (*n* = 216, 14.9%), contact investigation (*n* = 151, 10.8%), and TB in foreigners (*n* = 112, 7.9%).

**CONCLUSION:**

During the COVID-19 era, we received two-thirds of technical enquiries compared with 2017–2019, because local health offices were overwhelmed by the pandemic. Since the most frequent enquiries were about diagnosis and treatment of TB, the health ministry of Japan should maintain a few specialised TB institutions with TB physicians to provide technical assistance.

## Abstract (fr)

**CONTEXTE:**

Japon, un pays à faible taux de TB.

**OBJECTIF:**

Caractériser les demandes de renseignements techniques liées à la TB reçues en 2020–2022 et partager les enseignements tirés.

**MÉTHODE:**

Ceci est une étude descriptive.

**RÉSULTATS:**

Nous avons enregistré 1 898 communications, parmi lesquelles 1 447 (soit 40,2 par mois) ont été identifiées comme des demandes de renseignements techniques, ce qui représente une baisse de 34% par rapport aux 2 197 demandes reçues en 2017–2019. Les taux de demandes de renseignements étaient les plus élevés dans les préfectures de Shimane (4,32/100 000 habitants) et de Yamanashi (2,59/100 000 habitants), et les plus faibles dans les préfectures d'Ehime (0,00/100 000 habitants) et de Yamagata (0,09/100 000 habitants). Les principaux demandeurs étaient des médecins (*n* = 236 ; 16,3%), des infirmières (*n* = 814 ; 56,3%) et le grand public (*n* = 141 ; 9,7%). Les demandes les plus fréquentes concernaient le diagnostic et le traitement de la TB, y compris le diagnostic en laboratoire (*n* = 442 ; 30,6%), suivi du cadre réglementaire (*n* = 216 ; 14,9%), de la recherche des contacts (*n* = 151 ; 10,8%) et de la TB chez les étrangers (*n* = 112 ; 7,9%).

**CONCLUSION:**

Pendant la période de COVID-19, nous avons reçu deux tiers de demandes de renseignements techniques en comparaison avec 2017–2019, en raison de la surcharge des bureaux de santé locaux causée par la pandémie. Étant donné que les demandes de renseignements les plus fréquentes concernaient le diagnostic et le traitement de la TB, le ministère de la santé du Japon devrait maintenir quelques institutions spécialisées dans la TB avec des médecins tuberculeux pour fournir une assistance technique.

In Japan, the TB notification rate has decreased 85-fold over the past seven decades, from 698 per 100,000 population in 1951 to 8.2 per 100,000 population in 2022.^1,2^ However, around 4,000 cases of smear-positive pulmonary TB are reported annually, with over 75% involving persons aged 65 years or older.^3^ These potentially infectious TB cases pose a public health threat to the community, potentially causing TB outbreaks.^4–9^ To prevent and detect TB outbreaks early, local health offices are responsible for conducting contact investigations for cases under the Communicable Disease Control Act of 1998.^10–13^

The Research Institute of Tuberculosis (RIT) receives about 700 technical enquiries concerning TB each year from healthcare workers at local health offices and facilities nationwide, and the communications, including responses, are electronically stored on a computer server. These enquiries reflect the interests and concerns of frontline healthcare workers. However, detailed analysis has been limited, except for previous reports on enquiries received at RIT between 2014–2016 and 2017–2019, where 40–45% of the enquiries were related to the diagnosis and treatment of TB and TB contact investigations.^14,15^

As the number of TB cases declines in Japan, the number of TB experts may also decrease at a similar or faster rate. Additionally, during the COVID-19 era, Japan experienced 33 million cases and 74,000 deaths from COVID-19 between 2020 and 2023.^16^ Frontline healthcare workers were overwhelmed by managing COVID-19 patients and had little time to focus on TB patients. However, the National TB Programme (NTP) needs to retain clinical and epidemiological expertise in TB control to respond to enquiries.

This study aims to analyse and categorise the TB-related enquiries received by RIT in Japan from January 2020 to December 2022, corresponding to the COVID-19 era, and share the results, particularly with countries with a medium TB burden which may face similar challenges in the near future.

## METHODS

This is a descriptive study of TB-related enquiries in terms of time, place, and classification. We also analysed the correlation of the number of enquiries with the time they were received and the correlation of the rate of enquiries with TB notification rates of prefectures. The RIT receives enquiries and provides technical assistance on TB, including clinical and public health issues, through its website^17^ and by telephone. Announcements are made during training courses, stating that RIT welcomes enquiries and provides consultations. These courses accommodate more than 2,000 participants annually and are held at RIT, online, or locally with RIT staff attendance. Training content is adjusted according to the enquiries received daily.

When an enquiry is received by email or telephone at RIT, one of the primary responders (two physicians and three nurses) on duty replies. If further expertise is needed, the enquiry is forwarded to other staff members, including a consultant physician with expertise in TB, an epidemiologist, and two medical microbiologists. Summaries of telephone communications are recorded in a logbook. At the end of each month, an administrative staff member reminds all responders to record their telephone communications in the logbook.

An enquiry was defined as an event in which RIT received a telephone call or email containing a TB-related enquiry such as those related to diagnosis and treatment, including laboratory diagnosis and TB control, from January 2020 to December 2022.

A database of the enquiries has been established and maintained using Excel 2010 (Microsoft Corp, Seattle, WA, USA). The data recorded every month were extracted from email communications and the logbook recording telephone communications. Data included the date, the organisation of the enquirer, the profession of the enquirer, the initial classification of the enquiry, and a summary of the enquiry. To protect confidentiality, the names of the enquirers were not entered into the database. To avoid overrepresentation of enquiries, we classified one enquiry into two categories only if it explicitly included two independent queries and they were not strictly related. Two researchers (AY and MU) independently reviewed the database, particularly the classifications, and reclassified them if necessary. A third researcher (SH) decided the final classification if the decisions of the two researchers conflicted. If one or multiple follow-up communications were recorded, only the initial communication was counted if the follow-up communication(s) arrived within 1 month of the previous communication(s). Administrative, rather than technical, communications, such as requests to send a trainer for training sessions, conduct laboratory tests, or conduct interviews by media, were excluded from the analysis. Communications regarding non-tuberculous mycobacteria (NTM) were also excluded because the primary objective of the study is to provide background information for TB control in the country.

The correlations of enquiries by month and of enquiry rates and TB notification rates per 100,000 population among the prefectures were analysed using Pearson’s correlation coefficient. Student’s *t*-test was utilised for comparisons of means. R software (The R Foundation for Statistical Computing, Vienna, Austria) was used for these statistical tests. In calculating the enquiry rates and TB notification rates by prefecture, the 2020 census data (downloaded from the National Statistics Bureau of Japan) were used.^18^ The numbers of TB cases by prefecture were obtained from the routine surveillance data.^1^

### Ethics statement

We obtained a waiver of the ethical review for the study from the Institutional Review Board of the Research Institute of Tuberculosis (RIT/IRB 2023-30) because this study was retrospective and involved the secondary use of data already collected during routine operations.

## RESULTS

**FIGURE 1.**
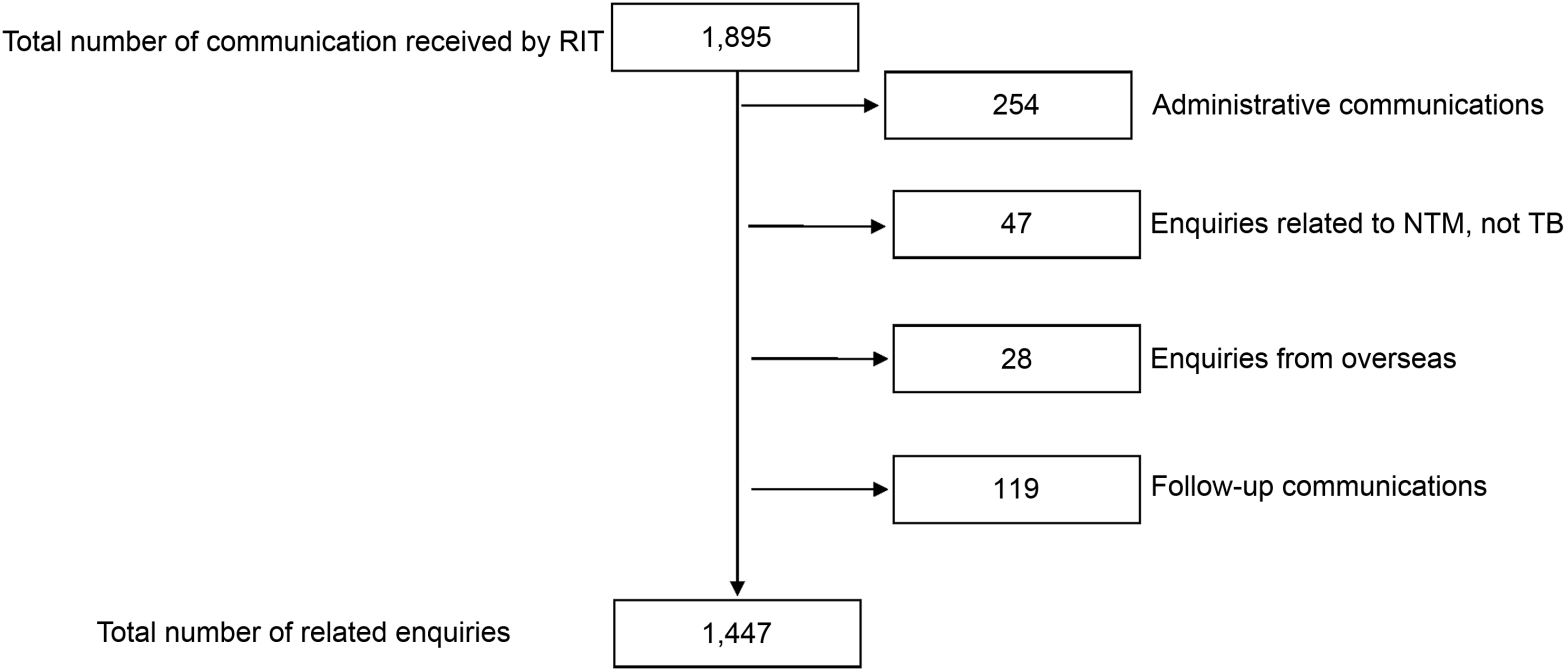
Schematic chart on the communications and the TB-related technical enquiries the RIT received, 2020–2022, Japan. NTM = non-tuberculous mycobacteria; RIT = Research Institute of Tuberculosis.

A total of 1,895 communications (2.5 per working day) were recorded, of which 1,447 (76.4%) were finally classified as TB-related technical ones. Some 254 enquiries were determined to be administrative, 47 were related to NTM, 28 were from overseas, and 119 were follow-up communications (Figure 1).

Communications were mostly received by email (*n* = 1,164, 80.4%); however, some enquiries were made by telephone (*n* = 283, 19.6%) (Table 1). The affiliations of the enquirers were local governments (*n* = 989, 68.3%), healthcare facilities (*n* = 242, 16.7%), and unknown/others (*n* = 216, 14.9%). A total of 741 different organisations were represented. Enquirers’ professions included medical doctors (*n* = 236, 16.3%) and public health and clinical nurses (*n* = 814, 56.3%) (Table 1).

**TABLE 1.** TB-related enquiries received by the Research Institute of Tuberculosis, Japan, 2020–2022.

	Enquiries
	*n* (%)
Total	1,447 (100.0)
In years	
2020	476 (32.9)
2021	490 (33.9)
2022	481 (33.2)
Way of communication	
Email	1,164 (80.4)
Telephone	283 (19.6)
Body enquirer belonged to:	
Local governments	989 (68.3)
Healthcare facilities	242 (16.7)
Unknown/other	216 (14.9)
Profession of enquirer	
Doctors	236 (16.3)
Nurses	814 (56.3)
Other healthcare workers	25 (1.7)
General public	14 (19.7)
Unknown/other	231 (16.0)

**FIGURE 2.**
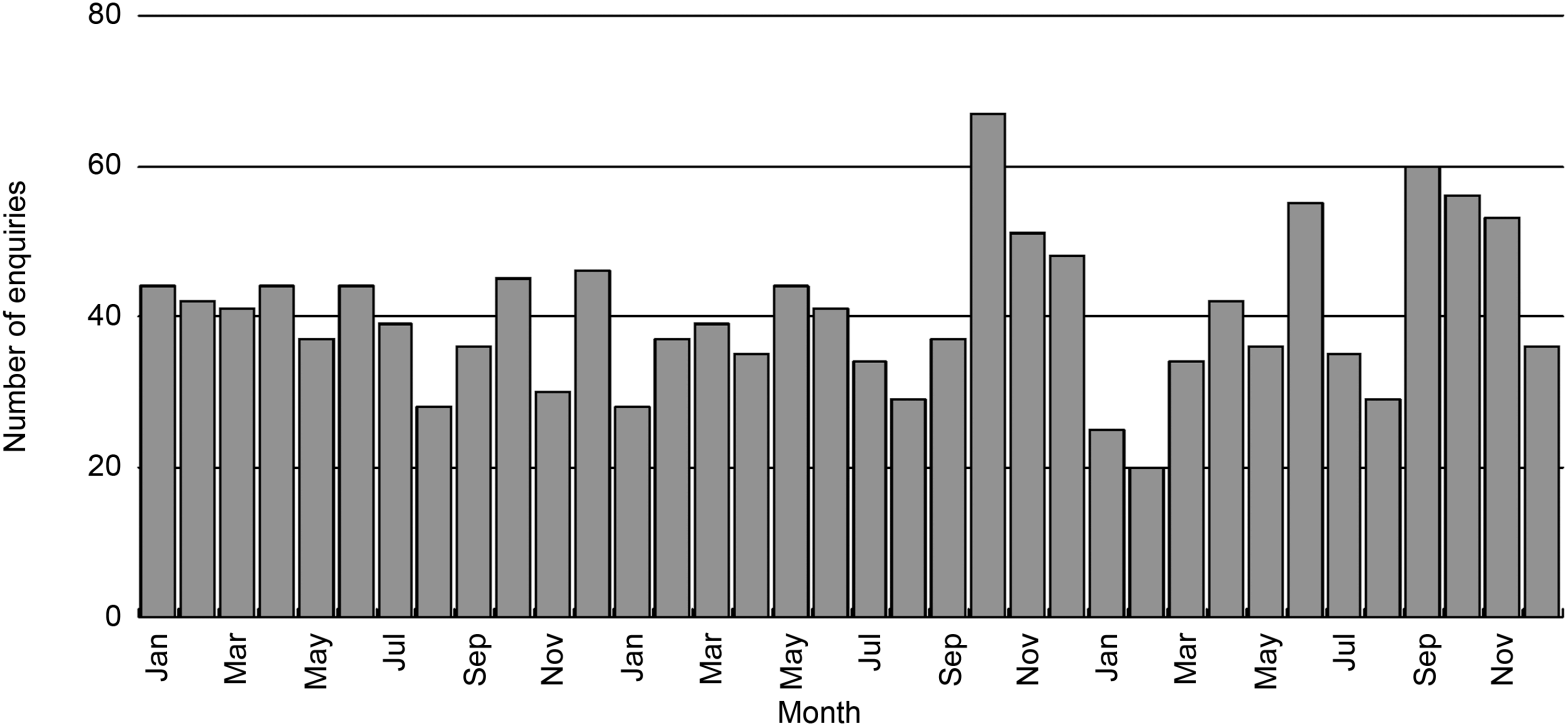
The number of TB-related enquiries received by month at the Research Institute of Tuberculosis, 2020–2022, Japan (*n* = 1,447; average = 40.2 enquiries/month; range = 20–67). No statistically significant increase or decrease in trend was observed (ρ = 0.17, 95% confidence interval [CI] −0.17 to 0.47).

We received an average of 40.2 enquiries (range 20–67) per month from January 2020 to December 2022, with no statistically significant increasing or decreasing trend (ρ = 0.17, 95% confidence interval [CI] −0.17 to 0.47) (Figure 2).

**FIGURE 3.**
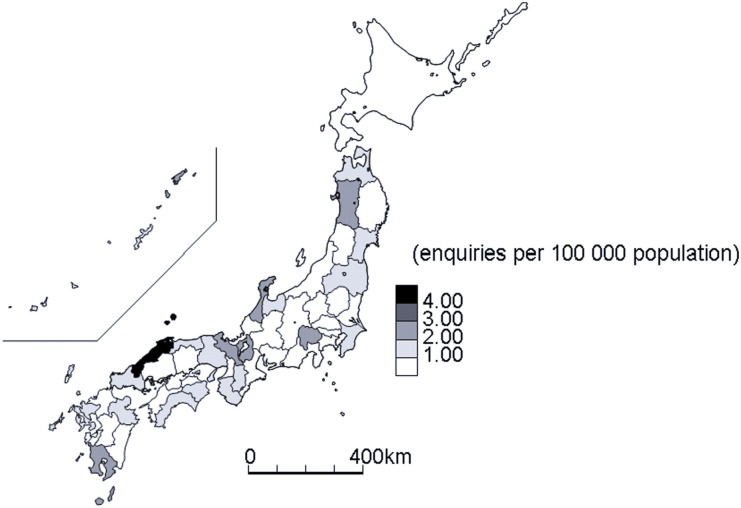
Geographic distribution of rates of TB-related enquiries received per 100,000 population at the Research Institute of Tuberculosis, 2020–2022, Japan.

The enquiry rate was highest (4.32/100,000) for Shimane, followed by Yamanashi (2.59/100,000) and Ishikawa (2.47/100,000) Prefectures, and was lowest in Ehime (0.00/100,000), followed by Yamagata (0.09 per 100,000 population) and Niigata (0.23/100,000) Prefectures (Figure 3). There was no correlation between the enquiry rates of the prefectures and their TB notification rates (ρ = 0.24, 95% CI −0.05 to 0.50).

The enquiries were related to TB diagnosis and treatment, including laboratory diagnosis (*n* = 442, 30.6%), regulatory framework (*n* = 216, 14.9%), contact investigations (*n* = 151, 10.8%), and TB in foreigners (*n* = 112, 7.9%) (Table 2). Enquiries related to TB diagnosis and treatment were further classified as TB diagnosis in general (*n* = 25, 1.7%), laboratory diagnosis (*n* = 68, 4.7%), anti-TB treatment in general (*n* = 27, 1.9%), management of comorbidities (*n* = 36, 2.5%), selection of anti-TB drugs when standard regimens cannot be used other than MDR-TB management (*n* = 39, 2.7%), treatment duration (*n* = 37, 2.6%), treatment of extrapulmonary TB other than TB pleuritis and TB in lymph nodes (*n* = 4, 0.3%), management of MDR- and rifampicin-resistant TB (*n* = 3, 0.2%), and others (*n* = 19, 1.3%).

**TABLE 2.** Classification of the TB-related enquiries received by the Research Institute of Tuberculosis, Japan, 2020–2022.

Classification of enquiries	Enquiries
	*n* (%)
Diagnosis/laboratory/treatment	442 (30.5)
Regulatory frame	216 (14.9)
Contact investigation	151 (10.4)
General inquiry	149 (10.3)
TB in foreigners/foreign countries	112 (7.7)
Latent TB infection	114 (7.9)
Bacille Calmette-Guérin	107 (7.4)
Dealing with TB patients	25 (1.7)
Health check-up	19 (1.3)
Others	112 (7.7)
Total	1447 (100.0)

**FIGURE 4.**
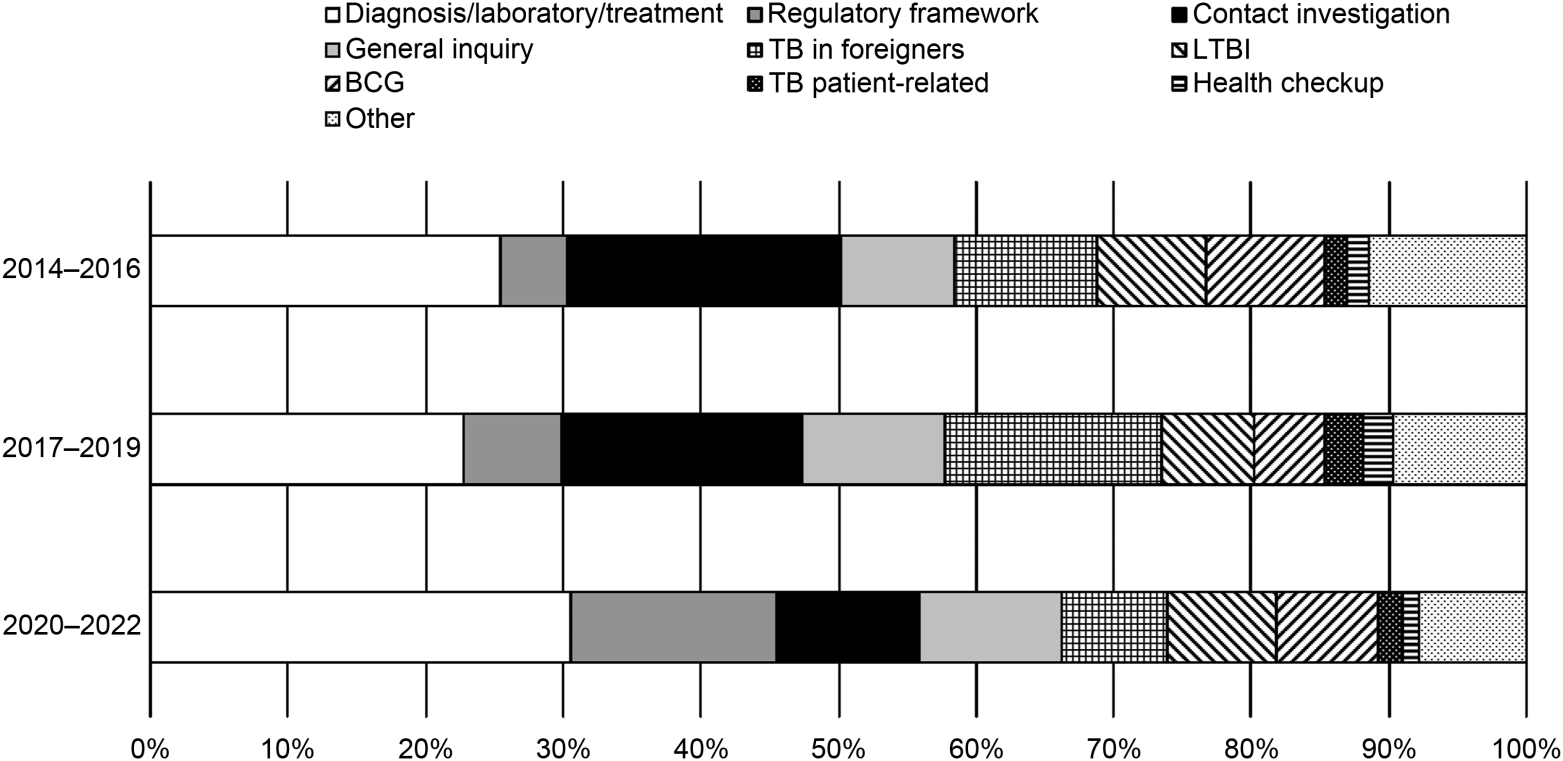
Trends in the categories of TB-related inquiries received at the Research Institute of Tuberculosis, 2014–2022, Japan.

We previously conducted similar studies on the enquiries on TB received in 2014–2016 and 2017–2019. We received 586, 630, 648, 743, 733, and 721 enquiries in 2014, 2015, 2016, 2017, 2018, and 2019, respectively (an average of 677.8 enquiries, 95% CI 609.2–744.5). Compared with the previous studies, the current study found a significantly lower number of enquiries received per year by 28.2% (677.8 vs. 482.3; *P* < 0.001). The proportions of each classification of the enquiries in 2020–2022 were compared with those of 2014–2016 and 2017–2019 (Figure 4). The proportion of the enquiries related to TB diagnosis and treatment is similar within the 20–30% range through the three study periods, whereas that of the regulatory framework suddenly increased from 4.8% in 2014–2016 to 7.1% in 2017–2019, to 14.9% in the current study. On the other hand, those related to contact investigations steeply decreased to 10.8% in the current study from 19.9% in 2014–2016 and 17.5% in 2017–2019.

## DISCUSSION

We conducted a review of enquiries about TB received domestically at RIT from 2020 to 2022. The majority of the enquiries concerned the diagnosis and treatment of TB, including laboratory diagnosis, the regulatory framework, and contact investigations. Almost two-thirds of the enquiries were from local governments, and only one-sixth were directly from healthcare facilities. There were substantial differences in the geographical distribution of enquiry rates by prefecture, ranging from less than 1/100,000 to >4/100,000.

One reason many enquiries were related to TB diagnosis and treatment is that general physicians, even pulmonologists, now have less expertise in diagnosing and treating TB patients than in the past because the number of TB patients has decreased, and the enquiries may not have been resolved at the local level.^19^ Frontline healthcare workers, including those at local health offices, may not have received regular training on TB, similar to the situation observed in the United States.^20^ Another reason is that over three-fourths of TB patients are elderly and more likely to present uncharacteristic signs and symptoms, causing difficulties in diagnosis, or to have adverse reactions to anti-TB medications because of relative frailty, the possible presence of multiple pathologies, and drug-drug interactions.^21–23^ Thus, physicians may need to deal with complicated situations that require expert advice.

It was surprising that the number and proportion of enquiries about the regulatory framework increased suddenly in 2020–2022, as RIT is not responsible for advising on the regulatory framework for TB; this is done by the Ministry of Health, Labour, and Welfare (MHLW). This is probably because, during the COVID-19 era, MHLW officials were too busy to respond to enquiries from local governments, who then turned to RIT as an alternative. Enquiries related to contact investigations became the third most common because it is sometimes difficult for public health professionals at local health offices to decide the scope of a contact investigation, requiring guidance from expert epidemiologists. As TB incidence declines, local health offices, particularly in cities with small to medium-sized populations of less than 1 million in their catchment areas, may not have many smear-positive TB cases in their jurisdictions and may lack experience in contact investigations, including outbreak investigations.

The substantial differences in the geographical distribution of enquiry rates in the prefectures might reflect the ease of access to local expert physicians rather than the actual TB notification rates in the prefectures. The number of enquiries decreased for 2020–2022 compared with 2014–2016 and 2017–2019 because local health offices were overwhelmed by the COVID-19 pandemic and may not have tried to resolve questions. Although limited to a few anecdotal examples, the number of contact investigations seems to have declined in 2020–2022, raising concerns that TB notification rates and the number of TB outbreaks may increase in the near future.

Our study has both strengths and limitations. One strength is that we characterised enquiries received over three consecutive years and compared the results with the previous two three-year periods. Thus, the results seem to represent TB enquiries at a national level and demonstrate changes over time. However, some basic enquiries were probably settled locally and may not be included in our study.^19^ The system of classifying enquiries might appear arbitrary, particularly the multiple categories of TB diagnosis and treatment, and of TB control; however, we have tried our best to accurately classify these enquiries, and we believe the classification reflects the reality of TB-related enquiries at the national level.

As most enquiries were related to TB diagnosis and treatment, MHLW of Japan should maintain a few specialised TB institutions with expert TB physicians and medical microbiologists available at the national or subnational level to provide technical assistance, including training, education, and medical consultation, to local governments and frontline healthcare workers.^20^ Additionally, the specialised TB institutions should provide technical assistance, including epidemiological support for TB contact and outbreak investigations, to local health offices requiring help, as done in the United States.^24^ Even during an infectious disease pandemic such as COVID-19 or potentially swine influenza, minimal resources should be allocated to TB control at local, provincial, and national levels as there are still about 4,000 cases of sputum smear-positive TB across Japan.
